# OA24 Airway obstruction in an adolescent: relapsing polychondritis

**DOI:** 10.1093/rap/rkac066.024

**Published:** 2022-09-28

**Authors:** Indhuja Rajarathinam, Nadia Rafiq, David D'Cruz

**Affiliations:** Guys and St Thomas Hospital, London, United Kingdom; Guys and St Thomas Hospital, London, United Kingdom; Guys and St Thomas Hospital, London, United Kingdom

## Abstract

**Introduction/Background:**

Relapsing polychondritis (RP) is a rare systemic autoimmune disease of unknown etiology, characterized by recurrent inflammation of cartilaginous and connective tissue. The disease includes chondritis of ears, nose, and larynx, evolving by recurrent flares, and may lead to floppy ears, saddle nose, and laryngotracheal stenosis. Very few cases have been reported in paediatrics with the most common presentation being auricular chondritis. Here, we report a patient who presented with acute airway obstruction requiring tracheostomy.

**Description/Method:**

A 16-year-old boy from Uganda, moved to UK 3 years ago and was referred to our department with Subglottic/tracheal stenosis of unknown aetiology. Initially it started as shortness of breath and then he developed voice hoarseness and stridor. CT neck showed severe laryngeal oedema and stenosis. He underwent an awake tracheostomy and pan endoscopy in theatre. He was noted to have severe laryngeal oedema and stenosis. Aetiology of lesion was unclear. He also reported significant weight loss.

Examinations at local hospitals had not identified the cause of the subglottic stenosis and he had not responded to antibiotic therapy. At this stage, he was referred to our hospital for a Rheumatological assessment. His condition evolved and he developed transient redness of his eyes and pain over his bilateral ears. On examination he had tender pinna with sparing of the lobule, a saddle nose with a transverse crease on his nose, and tenderness over the sternal region.

Laboratory findings showed increased inflammatory markers. Serology tests for antinuclear antibody and autoantibodies were all negative.

Chest computed tomography was normal. Biopsy specimens from Hypo pharyngeal and right subglottic region showed non-specific chronic inflammation and haemorrhage. PET CT was performed which showed increased uptake affecting the outer ears bilaterally and suggestion of low- grade uptake associated with the costal cartilages and more apparent xiphisternal uptake, suggestive of RP.

The patient fulfilled the McAdam-Damiani-Levine criteria for the diagnosis of RP, according to the presence of 3 of McAdam’s signs (auricular chondritis, ocular inflammation, and respiratory chondritis).

He was started on IV Methylprednisolone 10mg/kg/day for 3 days followed by oral steroids 40mg and tapered slowly. He was also started on Mycophenolate Mofetil 600mg/m2 twice daily, which led to an improvement in his symptoms. The auricular pain and pain over sternal region disappeared. No flare up to date.

**Discussion/Results:**

Paediatric-onset RP is rare and occurs in 5%–10% of the reported cases.

In almost all patients the presenting symptom is auricular chondritis. Arthralgia, with or without arthritis is the second commonest presenting symptom. Of note, neither of these symptoms were presenting feature in our patient.

Airway involvement while uncommon at presentation, occurs in approximately 50% of patients with RP. Airway manifestations are the commonest cause of morbidity and mortality in RP.

This patient was initially managed as asthma and was given bronchodilators with no clinical improvement. He then developed noisy breathing and dyspnoea which led to him being referred to a tertiary respiratory team. This is not unusual for the reported cases of RP that have initially been managed as asthma. The histological findings of chronic inflammation in the sub-glottis prompted a referral to Rheumatology.

In RP (unlike asthma), inhaled corticosteroids and bronchodilators are ineffective, spirometry demonstrates upper airway obstruction, CT imaging can reveal thickening and stenosis of the airways, and bronchoscopy can show inflammation, narrowing or collapse of the airways.

Currently there is no standard treatment for RP. No clinical trials have been carried out due to its rarity. Therefore, current medical therapy for RP is largely empirical and established on case reports. Despite this, RP generally responds to steroids and other immunosuppressive agents.

Corticosteroids are first-line therapy. They reduce severity and frequency of relapses. A low maintenance dose of steroid is used to prevent relapse and the dose is increased during flare ups. However, there is no evidence that corticosteroids alter the natural progression of RP. Steroid-sparing agents such as cyclophosphamide, methotrexate, Mycophenolate Mofetil, azathioprine and cyclosporine have been used and have shown benefit in the isolated reported cases. To date there is no evidence that one is more effective at preventing exacerbations than another.

**Key learning points/Conclusion:**

RP is a rare condition characterized by recurrent inflammation and destruction of cartilaginous structures.

RP can present with various clinical findings which can lead to a delay in diagnosis and management

The diagnostic criteria includes: McAdam et al.: (1) Recurrent chondritis of both auricles, (2) Nonerosive inflammatory polyarthritis, (3) Chondritis of nasal cartilages, (4) Inflammation of ocular structures, (5) Chondritis of respiratory tract, (6) Cochlear and/or vestibular damage  (Requirement—three out of six criteria)

Damiani and Levine: (1) Three out of six McAdam et al.'s criteria (2) One out of six McAdam et al.'s criteria and a positive histologic confirmation (3) Two out of six McAdam et al.'s criteria and response to corticosteroid or dapsone (requirement—any of these)

Asthma can mimic RP when the airways are involved.

Airway involvement in RP requires prompt corticosteroids/immunosuppression and if severe may lead to stenosis requiring emergency tracheostomy.

The treatment should be tailored according to individual patient's organ involvement and complications, although no guidelines exist to date.

Awareness of early airway involvement in RP is essential for prompt diagnosis and allows treatment to prevent life-threatening airway collapse.

It is essential to examine the auricle, nose and joints in patients presenting with atypical asthma not responding to conventional therapy.

